# Electroacupuncture Improves Cognitive Deficits through Increasing Regional Cerebral Blood Flow and Alleviating Inflammation in CCI Rats

**DOI:** 10.1155/2017/5173168

**Published:** 2017-04-10

**Authors:** Dexiong Han, Zhe Liu, Gaimei Wang, Ying Zhang, Zemin Wu

**Affiliations:** ^1^The 3rd Affiliated Hospital of Zhejiang Chinese Medical University, Hangzhou 310005, China; ^2^Zhejiang Chinese Medical University, Hangzhou 310053, China

## Abstract

*Objective*. To investigate the effect of EA on regional cerebral blood flow, cognitive deficits, inflammation, and its probable mechanisms in chronic cerebral ischemia (CCI) rats.* Methods*. Rats were assigned randomly into sham operation group (sham group) and operation group. For operation group, CCI model was performed using the permanent bilateral common carotid artery occlusion (BCCAO) method, and then rats were further randomly divided into model group and electroacupuncture (EA) group. 2/15 Hz low-frequency pulse electric intervention was applied at “Baihui” and “Dazhui” acupoints in EA group. Four weeks later, Morris water maze test was adopted to assess the cognitive function, using laser Doppler flowmetry to test changes of regional cerebral blood flow (rCBF); double antibody sandwich enzyme-linked immunosorbent assay (DAS-ELISA) to measure proinflammatory cytokines (IL-6, TNF-*α*, and IL-1*β*); western blot to test the protein expression quantities of proinflammatory cytokines, JAK2, and STAT3; and RT-PCR to test JAK2 mRNA and STAT3 mRNA in the hippocampus in each group.* Results*. Compared with the model group, learning and memory abilities and rCBF and IL-6 expression of the EA group enhanced markedly; IL-1*β* and JAK2 significantly decreased; TNF-*α* and STAT3 also declined, but the difference was not apparent.* Conclusion*. Our research suggests that EA can improve cognitive deficits which may be induced by increasing rCBF and anti-inflammatory effect.

## 1. Introduction

Long-lasting decrease in cerebral blood flow caused by chronic cerebral ischemia (CCI) is a key etiological factor of cognitive dysfunction among the aged [1–3]. The underlying pathological mechanisms behind CCI remain unclear. Previous studies have identified several neuropathological changes including immune inflammatory injury [4], oxidative stress injury [5], synaptic structural and functional disorders [6], neurotransmission disorders of the central cholinergic and monoaminergic system [7, 8], and white matter injury [9]. Permanent bilateral common carotid artery occlusion (BCCAO) model has been widely used in CCI because it can provide a moderate but persistent reduction in regional cerebral blood flow (rCBF) which compromises memory processes and contributes to the development and progression of various cognitive disorders [7].

Inflammation in the CNS occurs partly through the release of numerous factors like cytokines, chemokines, or growth factors, leading to a complex crosstalk between different brain cell types [10]. Inflammatory reaction, which is induced following the BCCAO in the white matter and hippocampus [3, 11], plays a crucial role in chronic ischemic cerebrovascular diseases [12]. The activation of JAK-STAT signaling pathway is an important component of the factors released during inflammation. Interleukin 1 (IL-1), IL-6, and tumor necrosis factor-alpha (TNF-*α*) are most widely studied [13] among the numerous inflammatory factors. Some of these inflammatory factors play synergistic roles in inflammatory injury, thereby aggravating cerebral ischemic injury and gradually deteriorating the cognitive function. Studies revealed that, during CCI injury process, electroacupuncture (EA) could upregulate vascular endothelial growth factor expression, promote the generation of new vessels, and improve neuronal function [14, 15]. However, these results are insufficient to elucidate the multitarget and multichannel mechanism of acupuncture. Thus, this study aims to investigate the potential mechanisms of EA on CCI from the aspect of increasing rCBF and preventing inflammation.

## 2. Materials and Methods

### 2.1. Animals

Animal care, operation, treatment procedures, and animal welfare were executed in strict accordance with the* National Institutions of Health Guide for the Care and Use of Laboratory Animals* by the Experimental Animal Center of Zhejiang Chinese Medical University. Healthy male Sprague–Dawley (SD) rats (weight 200 ± 20 g and ages 3–6 months) were brought from and fed in the Experimental Animal Center of Zhejiang Chinese Medical University. They were placed in standard cages under a specific circumstance where light-dark cycle is 12 h/12 h, relative humidity is 45%–50%, and temperature is 22 ± 2°C. The enrolled rats first underwent the Morris water maze test to exclude those with low intelligence and poor swimmers to ensure that the cognitive function did not show any significant differences among all rats before the study.

### 2.2. Animal Modeling and Evaluation

*Operation Group*. CCI model was performed by BCCAO method [16]. In brief, under 10% chloral hydrate (40 mg/kg, i.p.) anesthesia, bilateral common carotid arteries of the rat were carefully separated from the cervical sympathetic and vagal nerves through a ventral cervical incision. Then, the arteries were doubly ligated with 5/0 silk sutures simultaneously. The skin incision was closed and the rats were kept in an air-conditioned room as described previously. Before the operation, the rats were fastened for 12 h and deprived of water for 4 h.

*Sham Group*. The rats were treated in the same manner as the operation group except for the bilateral common carotid artery occlusion.

Two hours after the rat recovered from anesthesia, two rats were randomly selected from respective operation group and sham operation group to evaluate rCBF and then killed and their brains were sliced for pathological evaluation to ensure that the rat model of CCI was successfully copied.

### 2.3. Grouping

After modeling, rats of the operation group were further assigned randomly into the model group and EA group, each consisting of 10 rats. The sham group also comprised 10 rats.

### 2.4. EA Treatment

Rats of the EA group receive EA therapy. In the whole procedure, slip eyeshades over rats' eyes to keep them relatively comfortable and quiet. “Baihui” (DU20) and “Dazhui” (DU14) acupoints were selected according to the* Acupoint Standard for Experimental Animals* issued by the Society of Experimental Acupuncture, China Association for Acupuncture and Moxibustion in 1992. Acupuncture was performed using the following instruments and parameters: needle: 0.25 mm in diameter × 25 mm in length Huatuo stainless steel filiform needle (Suzhou Medical Supplies Co., Ltd., China) and HAN's Acupoint Nerve Stimulator (Nanjing Jisheng Medical Treatment Technology Co., Ltd., China): 2/15 Hz dilatational wave of frequency with automatically shifting between 2 Hz and 15 Hz stimulation for 3 s each, with current intensity 2 ± 1 mA (causing slight vibration of muscles around acupoints and keeping quiet), lasting for 30 min, once in the morning, five times every week, with break at weekend, and the treatment continued for 4 weeks.

### 2.5. Evaluation Indicators and Testing Methods

#### 2.5.1. Evaluation of Cognitive Function

Cognitive function of various groups was evaluated by Morris water maze system (Beijing Taiji Electronic Co., Ltd., Beijing, China).

*(1) Navigation Test*. The test started from the last week of the treatment. After EA therapy was completed in the EA group, rats of the three groups underwent a navigation test, twice daily, with an interval of 15 min, for successive 5 days, and the average values were adopted. Experimental data and images were analyzed using an image automatic monitoring and processing system.

*(2) Probe Trial*. The trial was conducted on the fifth day after conducting the navigation test. The platform in the pool was removed, and the rats were allowed to swim freely, where the entrance was located at the contralateral quadrant of the platform. The memory function was evaluated using the time for the rats to cross the quadrant of the former platform within 120 s. This trial was performed twice, with an interval of 15 min.

#### 2.5.2. rCBF Test

rCBF was tested before and after BCCAO (within 30 min) and before killing by laser Doppler flowmetry (PeriFLUX5000, Sweden) including the following steps. Right temporal side of the brain of rats [14] (midpoint of the outer canthus and the earhole) was selected as the testing point. Hair was shaved, skin was disinfected with alcohol and polyvinyl iodine, and then a 5 mm longitudinal incision was made. The muscles were bluntly disserted to expose the bone surface, which was cleaned with sterile cotton balls dipped in 3% H_2_O_2_. Then the probe base was fixed onto the bone surface using specific glue. After this, the probe was inserted into the base, and these data were recorded and stored after they were stabilized on the computer monitor (recording time ≥ 3 min). Rats were anesthetized with ether in specimen jar for about 60–90 seconds to be just unconscious before test each time.

#### 2.5.3. DAS-ELISA

Rats were rapidly sacrificed after anesthesia with chloral hydrate after the last rCBF test. Concentrations of IL-6, TNF-*α*, and IL-1*β* in the hippocampus of rats were determined by DAS-ELISA according to the manufacturer's instructions (Shanghai Westang BioTech Inc., Ltd., Shanghai, China).

#### 2.5.4. Western Blot

Western blot was performed to measure protein expression of IL-6, TNF-*α*, IL-1*β*, JAK2, and STAT3. Hippocampus tissues were homogenized in strong RIPA buffer. The homogenate was allowed to rest on ice for 30 min and then centrifuged at 15,000 rpm for 15 min at 4°C, and the supernatant was collected. The protein concentration of tissue lysates was determined with a BCA protein assay kit. The lysates were separated on 10% SDS-PAGE gel and transferred to polyvinylidene difluoride (PVDF) membranes (Bio-Rad, Hercules, CA, USA). The membranes were blocked with 5% nonfat powdered milk in TBST (Tris-buffered saline containing 0.1% Tween 20) for 1 h at room temperature (RT) and then incubated overnight at 4°C with the following primary antibodies: IL-6 (Abcam); IL-1*β* (Millipore); TNF-*α* (Santa Cruz); JAK2 (Sigma-Aldrich), STAT3 (Sigma-Aldrich), *β*-actin (Abcam). After washing in TBST, the membrane was incubated for 1 h at RT with HRP-conjugated goat anti-rabbit antibody (West Grove), and protein bands were visualized using the Immun-Star™ HRP Chemiluminescence Kit (Bio-Rad). Images of bands were recorded by the ImageQuant LAS 4000 system (GE Healthcare) and the band intensities were quantified using ImageQuant TL software (version 7.0, GE Healthcare). *β*-Actin was used as the internal loading control.

#### 2.5.5. RT-PCR

Total RNA was isolated from hippocampus by TriBlue RNA kit (Ambion, USA) according to the manufacture's protocol. 1 *μ*g of total RNA was used as a template for reverse transcription using. PCR amplification of the JAK2, STAT3, and *β*-actin was as follows: JAK2, sense: 5′-GTTCTTACCGAAGTGCGTGCGA-3′, antisense: 5′-GGTAATGGTGTGCATCCGCAGTT-3′; STAT3, sense: 5′-TGGAAGAGGCGGCAGCAGATAGC-3′, antisense: 5′-CACGGCCCCCATTCCCACAT-3′; *β*-actin, sense: 5′-TCAGGTCATCACTATCGGCAAT-3′, antisense: 5′-AAAGAAAGGGTGTAAAACGCA-3′. The thermal cycling parameters were as follows: JAK2, 95°C for 5 min, followed by 30 cycles of amplification at 95°C for 20 s, and 72°C for 45 s; STAT3, 95°C for 5 min, followed by 30 cycles of amplification at 95°C for 20 s, and 60°C for 30 s; *β*-actin, 95°C for 5 min, followed by 30 cycles of amplification at 94°C for 30 s, and 72°C for 30 s. The amplification products were incubated with EB, separated from agarose gel electrophoresis, and detected by GIS Gel image processing system. The average optical density of the bands was analyzed with *β*-actin as internal control.

### 2.6. Statistical Analysis

Statistical analyses were performed by SPSS 16.0 (SPSS, IL, USA). Descriptive data were expressed as mean ± standard deviation ($\overset{-}{x}\pm s$). For the navigation test, multiple-factor variance analysis for repeated measurement data was adopted. Intragroup comparisons were performed before and after intervention using pairwise *t*-test and intergroup comparisons were conducted using one-way analysis of variance (ANOVA). In addition, the *t*-test was applied after the data were confirmed with equal variance while the rank-sum test was adopted after the data were confirmed with unequal variance. A *P* value of less than 0.05 was considered statistically.

## 3. Results

### 3.1. Evaluation of Cognitive Function

#### 3.1.1. Navigation Test

**Figure 1:**
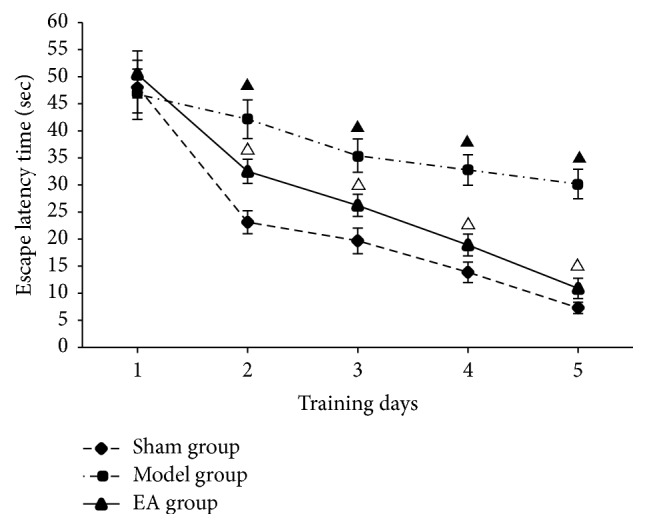
Performance of rats during navigation test. ^▲^*P* < 0.01, versus sham group; ^△^*P* < 0.05, versus model group.

Navigation test results showed that although escape latency did not have a significant difference among three groups on the first day (*P* > 0.05), after 5-day training, compared with model group, the escape latency of the sham group quickly decreased to a plateau (*P* < 0.01), while that of EA group also significantly declined to a plateau (*P* < 0.05), indicating that EA can improve learning and memory ability in CCI rats (Table 1, Figure 1).

#### 3.1.2. Probe Trial

**Figure 2:**
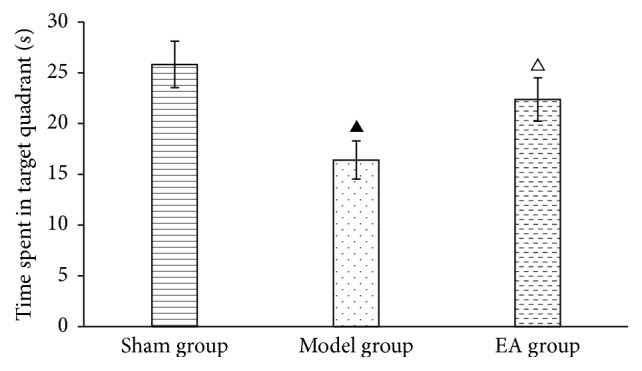
Time spent in the target quadrant of rats. ^▲^*P* < 0.05, versus sham group; ^△^*P* < 0.05, versus model group.

Time spent in the plateau quadrant of the EA group was longer than that of the model group, with a significant difference between two groups (*P* < 0.05), and close to that of the sham group, suggesting that EA can improve the memory in CCI rats (Table 2, Figure 2).

### 3.2. Changes of rCBF

#### 3.2.1. rCBF before/after BCCAO

The rCBF of pre-BCCAO did not show any significant difference between the sham and operation group (*P* > 0.05), while there was significant decrease (*P* < 0.01) in operation group after BCCAO, as shown in Table 3.

#### 3.2.2. rCBF after EA

After receiving the 4-week treatment, the rCBF of the EA group significantly increased compared with the model group (*P* < 0.05), though it is still lower than sham group (*P* < 0.05), as shown in Table 4.

### 3.3. Effects of EA on IL-6, TNF-*α*, and IL-1*β* ($\overset{-}{x}\pm s$, ng/mL)

**Figure 3:**
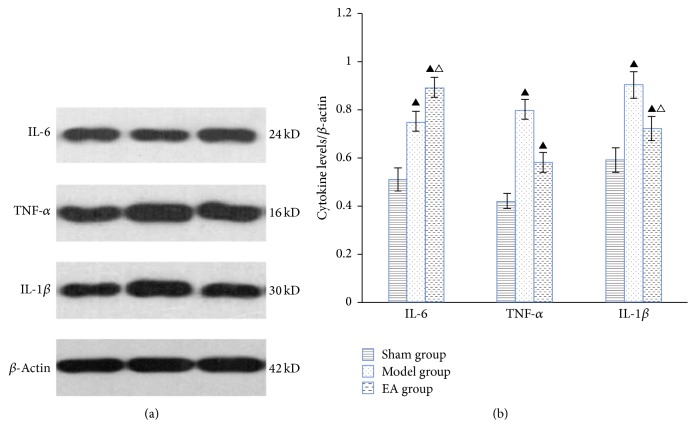
The protein levels of IL-6, TNF-*α*, and IL-1*β* in the hippocampus. (a) Representative western blots of proinflammatory cytokines. *β*-Actin was used as the loading control. (b) Quantitative analysis of cytokine expression/*β*-actin. ^▲^*P* < 0.05, versus sham group; ^△^*P* < 0.05, versus model group.

After the 4-week treatment, the IL-6 expression of the model and EA groups increased compared with the sham group, and the difference was statistically significant (*P* < 0.05). Moreover, it increased more remarkably in the EA group, with a significant difference compared with the model group (*P* < 0.05). Also, the expression of TNF-*α* and IL-1*β* in the EA and model group increased compared with the sham group; however, they declined in EA group compared with the model group. Particularly, the IL-1*β* decreased more obviously with a significant difference between EA group and model group (*P* < 0.05) (Table 5 and Figure 3).

### 3.4. Effects of EA on JAK2 and STAT3

**Figure 4:**
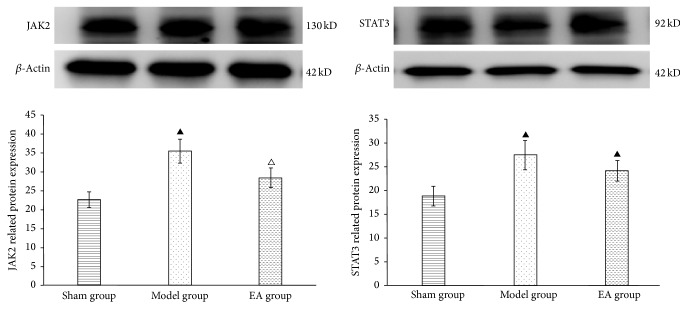
Expression of JAK2 and STAT3 protein by western blot in each group. ^▲^*P* < 0.05, versus sham group; ^△^*P* < 0.05, versus model group.

Results showed that 4 weeks after BCCAO, expression of JAK2 and STAT3 mRNA enhanced in model and EA group, particularly in the former; there was significant difference between sham group and model group (*P* < 0.05). However, they were declined in EA group, especially the JAK2 mRNA; there was significant difference between EA group and model group. Western blot showed that expression of JAK2 and STAT3 enhanced in model and EA group, particularly in the model group; there was significant difference between sham group and model group (*P* < 0.05). But they were declined in EA group, especially JAK2; there was significant difference between EA group and model group as shown in Table 6 and Figure 4.

## 4. Discussion

CCI is common in elderly society, which has been identified as a notable risk factor of dementia in patients with cerebrovascular disease [17, 18]. It is caused by long-term or chronic cerebral hypoperfusion, and it manifests as a group of persistent or progressive cognitive disorders. It is the common pathological basis of vascular dementia, Alzheimer's disease, Binswanger's disease, and so on [1]. Published studies have revealed that individual spatial learning and memory abilities can be achieved only by mutual cooperation of different brain regions, such as hippocampus, striatum, and cerebellum; reduction of CBF in these regions correlates directly with cognitive impairments [19]. The hippocampus plays a crucial role and its neurons are considered as physiological basis of spatial learning and memory abilities [20]. CCI induces long-term and persistent cerebral hypoperfusion. Thus, the energy metabolism of the hippocampus and other sensitive areas may not be improved continuously, thereby leading to the progressive aggravation of cognitive dysfunction [19]. Some studies reported a diffusely decreased blood flow in the frontal lobe, hippocampus, and other important brain regions 2 years before cognitive function symptoms appear in patients with vascular dementia [21]. Detecting the cerebral blood flow in SD rats of the BCCAO model revealed that the blood flow in the brain cortex and hippocampus significantly decreased 2.5 h after ischemia. Moreover, it most remarkably decreased in the hippocampus, with a decrease in rate up to 60% [22]. Meanwhile, with the gradual extension of cerebral hypoperfusion, the neurons in the sensitive areas might gradually appear with ischemia, edema, degeneration and necrosis, depigmentation, and other pathological changes [23]. As reported, this study also showed that the rCBF of the operation group significantly decreases after BCCAO, and the control level dropped sharply and reduced to 35–45% in the cerebral cortex and 60% in the hippocampus in the acute phase. Then, the CBF values started to increase gradually from the first week, which is a phase of chronic hypoperfusion closely resembling human, but it remained significantly lower than sham group [7]. After receiving the 4-week EA treatment, as showed in other studies [24], the rCBF of EA group improved statistically compared with the model group, though it was still lower than that of the sham group.

Inflammatory response is well documented and plays a key role in CCI injury [11, 25], which includes inflammatory immune cells, inflammatory immune factors, and adhesion molecules. Among inflammatory immune factors, IL-1*β*, TNF-*α*, and IL-6 are closely associated with the central nervous system and ischemic injuries, which can lead to demyelination, oligodendrocyte apoptosis, thrombosis, vascular proliferation, leukocyte infiltration, and blood-brain barrier disruption [26]. Of these, IL-1 is an important inflammatory factor triggering immune and inflammatory response, which also can aggravate ischemic brain injury. Particularly, IL-1*β* is closely associated with the pathogenesis of cerebral ischemia [27]. IL-1*β* also has a synergistic effect with TNF-*α* to further exacerbate brain damage [28, 29] and cerebral ischemia can upregulate TNF-*α* and participate in the pathological process of brain injury [30], while TNF-*α* can promote the release of IL-1*β* and other cytokines. IL-6 plays a complex role in the central nervous system; its role in cerebral ischemic injury is still controversial [31, 32]. Some believed that IL-6 is beneficial in cerebral ischemia [33], but some deemed it may exacerbate brain injury [32]. However, the majority of available studies indicate that IL-6 exerts a neuroprotective effect and promotes the recovery of neurological functions. Inflammatory response can be propagated and amplified by the JAK-STAT signaling pathway [34]. JAK-STAT signaling has been reported to be involved in inflammatory responses [35]. As JAK2 is upstream STAT3, present study shows that although normal levels of JAK2/STAT3 activation are essential for cellular functions, excess STAT3 activation is detrimental to brain, and prevention of ischemic-induced JAK2/STAT3 phosphorylation is neuroprotective [36]. The findings of our study also revealed that EA could modulate the expression of IL-1*β*/TNF-*α*/IL-6 and downregulate the excess activation of JAK2/STAT3 in CCI rats.

EA has been proved to be capable of improving cognitive function, which might be associated with regulating cell proliferation in different brain regions [15] and activating adenosine monophosphate-activated kinase (AMPK) [37]. In addition, EA showed anti-inflammatory effects including attenuating the activation of microglia via the TLR4/NF-*κ*B signaling pathway [38] and the TLR2, JNK/c-Jun pathways, modulating the release of TNF-*α*, IL-1, and IL-6 in MACO rats [39]. Our research was consistent with it. Results showed that EA on DU20 and DU14 is prone to improve cognitive function modulating the release of inflammatory immune factors including TNF-*α*, IL-1*β*, and IL-6 which may be achieved through improving the rCBF and depressing the excess activation of JAK2/STAT3 signaling pathway.

Of course, until now, only initial conclusions have been drawn and we will do further research upon the following goals: firstly, classical CCI model was evaluated by the water maze and pathological section in 3–6 weeks after BCCAO operation and this study emphasizes intervention in early stages. Therefore, in addition to rCBF, it is imperative to find a new evaluation method. Secondly, it is meaningful to investigate the effect of EA on phosphorylation of JAK2/STAT3 pathway. Thirdly, increasing a blocking group and blocking the JAK/STAT pathway with inhibitor AG490 are necessary to clarify the effect of EA.

## 5. Conclusions

In summary, our present evidence demonstrated that EA on DU20 and DU14 can improve cognitive function after CCI. This may be achieved by increasing rCBF, downregulating the excess activation of JAK2 and STAT3, and mediating IL-6, TNF-*α*, and IL-1*β* levels thus attenuating inflammatory injury. Though the protective mechanisms have not been fully elucidated, the data demonstrate that EA is a promising approach to treat chronic cerebral ischemia.

## Figures and Tables

**Table 1:** Performance of rats during navigation test ($\overset{-}{x}\pm s$, s).

Groups	Training days				
	1	2	3	4	5
Sham group	48.17 ± 4.87	23.11 ± 2.12	19.67 ± 2.38	13.87 ± 1.89	7.29 ± 1.05
Model group	46.76 ± 4.65	42.15 ± 3.57^a^	35.42 ± 3.08^a^	32.77 ± 2.82^a^	30.18 ± 2.73^a^
EA group	50.43 ± 4.35	32.51 ± 2.23^b^	26.23 ± 2.05^b^	18.92 ± 2.01^b^	10.89 ± 1.87^b^

Compared with the sham group, ^a^*P* < 0.01; compared with the model group, ^b^*P* < 0.05.

**Table 2:** Time spent in the target quadrant ($\overset{-}{x}\pm s$).

Groups	Time spent in plateau quadrant (s)
Sham group	25.82 ± 2.29
Model group	16.41 ± 1.88^a^
EA group	22.37 ± 2.13^b^

Compared with sham group, ^a^*P* < 0.05; compared with model group, ^b^*P* < 0.05.

**Table 3:** Changes of rCBF before/after BCCAO ($\overset{-}{x}\pm s$, PU).

Groups	Before BCCAO	After BCCAO
Sham group	160.85 ± 14.08	155.89 ± 13.82
Operation group	157.57 ± 13.93	63.15 ± 8.06^a^

Compared with the sham group, ^a^*P* < 0.01.

**Table 4:** Changes of rCBF in various groups ($\overset{-}{x}\pm s$, PU).

Groups	rCBF
Sham group	156.38 ± 14.29
Model group	89.05 ± 8.18^a^
EA group	125.89 ± 10.38^bc^

Compared with the sham group, ^a^*P* < 0.01; compared with the model group, ^b^*P* < 0.05; compared with the sham group, ^c^*P* < 0.05.

**Table 5:** Changes of IL-6, TNF-*α*, and IL-1*β* in the hippocampus ($\overset{-}{x}\pm s$, ng/ml).

Groups	IL-6	TNF-*α*	IL-1*β*
Sham group	0.305 ± 0.019	0.118 ± 0.022	0.223 ± 0.021
Model group	0.331 ± 0.015^a^	0.170 ± 0.012^a^	0.336 ± 0.026^a^
EA group	0.372 ± 0.011^ab^	0.148 ± 0.020^a^	0.279 ± 0.037^ab^

The protein levels of IL-6, TNF-*α*, and IL-1*β* were determined by ELISA. Compared with the sham group, ^a^*P* < 0.05; compared with the model group, ^b^*P* < 0.05.

**Table 6:** Expression of JAK2/STAT3 mRNA ($\overset{-}{x}\pm s$).

Groups	JAK2	STAT3
Sham group	1.125 ± 0.164	0.795 ± 0.158
Model group	1.528 ± 0.182^a^	1.152 ± 0.164^a^
EA group	1.292 ± 0.162^b^	1.002 ± 0.171^a^

The mRNA levels of JAK2 and STAT3 were detected by RT-PCR. Compared with sham group, ^a^*P* < 0.05; compared with model group, ^b^*P* < 0.05.
